# Abatement of Azo Dye from Wastewater Using Bimetal-Chitosan

**DOI:** 10.1155/2013/476271

**Published:** 2013-11-20

**Authors:** Ghorban Asgari, Bahman Ramavandi, Sima Farjadfard

**Affiliations:** ^1^Department of Environmental Health Engineering, Faculty of Health, Hamadan University of Medical Sciences, Hamadan, Iran; ^2^Department of Environmental Health Engineering, Faculty of Health, Bushehr University of Medical Sciences, Bushehr 7518759577, Iran; ^3^Department of Environmental Engineering, Graduate School of the Environment and Energy, Science and Research Branch, Islamic Azad University, Tehran, Iran

## Abstract

We introduce a new adsorbent, bimetallic chitosan particle (BCP) that is successfully synthesized and applied to remove the orange II dye from wastewater. The effects of pH, BCP quantity, and contact time are initially verified on the basis of the percentage of orange II removed from the wastewater. Experimental data reveal that the Cu/Mg bimetal and chitosan have a synergistic effect on the adsorption process of the adsorbate, where the dye adsorption by Cu/Mg bimetal, chitosan alone, and bimetal-chitosan is 10, 49, and 99.5%, respectively. The time required for the complete decolorization of orange II by 1 mg/L of BCP is 10 min. The Langmuir model is the best fit for the experimental data, which attains a maximum adsorption capacity of 384.6 mg/g. The consideration of the kinetic behavior indicates that the adsorption of orange II onto the BCP fits best with the pseudo-second-order and Elovich models. Further, the simulated azo dye wastewater can be effectively treated using a relatively low quantity of the adsorbent, 1 mg/L, within a short reaction time of 20 min. Overall, the use of BCP can be considered a promising method for eliminating the azo dye from wastewater effectively.

## 1. Introduction

It is well known that azo dyes are recalcitrant and toxic contaminants to aquatic organisms and carcinogenic and mutagenic to humans [1]. Therefore, in order to avoid the environmental problems caused by these pollutants and their hazardous effects on living beings, it is necessary to find technologies that effectively remove azo dyes from textile wastewater before discharging this effluent into the water resources.

Many researchers have evaluated the effectiveness of several types of low-cost biomaterials, such as chitosan [2], cellulose [3], and *Rhizopus oryzae* [4], for the removal of various dyes. Chitosan, poly (1→4)-2 amino-2-deoxy-*β*-D-glucan, is usually obtained from waste biomass during seafood processing and mainly comprises shells of crabs, shrimp, prawns, and krill [5]. Various biomaterials based on chitosan have already been tested as excellent adsorbents for the adsorption of various dyes from aqueous solutions. Chitosan has three functional groups, namely, two hydroxyl groups (–OH) and one amino group (–NH_2_), per glucosamine unit [5]. According to literatures reviewed by Crini and Badot [6], the critical challenges in this field remain low efficiency and the leaching of toxic adsorbates into the environment due to the weakness mechanisms involved in adsorption. These challenges technically and environmentally restrict adsorbents full-scale application. Therefore, the main concern related to azo dyes is the development of a more efficient adsorbent with a simple and low-cost production method. This type of adsorbent would lead to the removal of a higher amount of pollutants in the reactor while enhancing the rate of decontamination of the target contaminant(s) at a lower cost. The modification of biomaterials aims at improving their adsorption capability, thus reducing the rate of material consumption in order to lower the cost of the adsorption process making it more cost-effective. Ongoing research has attempted to find novel methods to enhance the adsorbent capacity of these materials while finding adsorbents that are easy to use and economical to produce in order to expand their utility as industrial biomaterials.

The chemical structure of chitosan offers an opportunity to trap the bimetallic particles, and the property of high water solubility [5] makes it possible for the bimetal-chitosan to effectively remove azo dyes without significant shaking and energy consumption.

Bimetallic particles enhance metal-pollutant reactivity and may be obtained by coating a small amount of less active metals, such as Pd, Ni, Pt, and Cu, onto fresh metal surfaces, which promote metal oxidation given the potential difference. In this study, we investigated the use of an innovative method, such as the use of bimetallic Cu/Mg particles consisting of a core metal (Mg) and a second metal (Cu) for removing the azo dye from a solution. Several researchers have successfully applied bimetallic particles for the remediation of pollutants, such as organic chlorinated hydrocarbons [7], polychlorinated biphenyls [8], nitrate [9], heavy metals [10], and azo dyes [11]. Although considerable research has been conducted on azo dye decolorization by zero-valent iron and bimetallic particles with Fe^0^-core metal, few practical technologies have been applied to use them given their complex nature and the many challenges that they pose when compared to other metals, such as Mg^0^, including the need for a higher dose and the possibility of Fe^2+^ entering the water to block the reaction by Fe(OH)_3_ precipitation, thereby inducing a red color into the solution and exhibiting a low standard potential [11]. On the other hand, magnesium, one of the most abundant elements in the crust of the earth and the seawater, is environmentally acceptable (nontoxic) with a high hydroxide solubility; the Mg^0^ surface is not inactive given the precipitation of Mg(OH)_2_ during treatment [10]. Accordingly, zero-valent magnesium has a better potential for azo dye removal than iron.

Therefore, in the present study, we focus on the preparation, characterization, and adsorption properties of bimetallized chitosan particles, as a novel adsorbent, for a model pollutant. An azo dye, orange II (OII), was chosen as the model pollutant because of its anionic character and its extensive use in the textile industry as well as in some limited medical diagnosis applications. This report presents the first application of bimetallic chitosan particle (BCP) for the removal of azo dyes from wastewater. The influences of the following basic variables were evaluated in the tests on the adsorption of OII, solution pH, BCP concentration, pollutant concentration, and reaction time. Evaluations were also undertaken to elucidate the kinetic behavior of the considered BCP and the mechanism of OII adsorption onto BCP. For the latter, the applicability of BCP was investigated in the treatment of the OII-spiked river water (as a sample of the azo-rich wastewater) under optimized conditions.

## 2. Materials and Methods

### 2.1. Materials

With the exception of the dye, all the chemicals used in these experiments were of analytical grade and purchased from Merck (Darmstadt, Germany). The OII dye was purchased from Fluka (Buchs, Switzerland). The dye content was 85%, and the solutions were prepared accordingly. The main properties of OII are presented in Table 1 [12, 13]. The produced chitosan was a meso- and macroporous material. The main properties of the BCP are shown in Table 2. Double distilled water was used for preparing all the solutions.

### 2.2. Extraction of Chitosan from Shrimp Waste

The shrimp shell waste derived from *Philocheras lowisi* was directly collected from the Persian Gulf, Iran, in September 2012. The shrimps were placed on ice during their transfer to the laboratory, and the species *Philocheras lowisi* was selected for this study. In particular, 700 shrimps were deshelled for the extraction of the chitosan. First, the shells were cleaned, rinsed, and then submerged in 10 wt.% NaOH for 2 h with agitation to remove proteins (20% w/v), in 1.8 mol/L of HCl for 12 h to remove calcium minerals (25% w/v), and in 0.38 wt.% NaClO for 0.5 h with agitation to remove the pigments (25% w/v). The product, chitin, was deacetylated in 50 wt.% NaOH for 1 h at 110°C (15% w/v). The deacetylation above 65% in experiments performed at a slightly lower temperature and shorter contact times was similar to the conditions reported by No and Meyers (1995) [14] and Novikov (2004) [15]. The mixture was then washed with distilled water several times to remove the residual sodium hydroxide, until a pH of 7.5 was achieved. The product, chitosan, was dried at 50°C for 8 h and finally sieved for modification by bimetal particles.

### 2.3. Bimetallic Chitosan Preparation

Chitosan modified by Cu/Mg particles was prepared by using a modified water-based approach [16]. The preparation was performed in a 250 mL flask attached to a vacuum line. In particular, a given amount of chitosan particles was immersed in double distilled water and was then purged with purified N_2_ for 45 min in order to remove the dissolved oxygen. In a typical preparation, first, a stock solution of 0.21 M MgCl_2_·6H_2_O was prepared right before use and then added to the chitosan solution to yield the desired concentration of Mg^2+^ and chitosan. The mixture was purged with N_2_ in an ultrasonic bath for 1 h to ensure the complete formation of the Mg^2+^-chitosan complex. Second, the Mg^2+^ ions were reduced to Mg^0^ by adding a certain amount of sodium borohydride (BH_4_^−^/Mg^2+^ = 2.0) dropwise to the above Mg^2+^-chitosan solution under inert conditions through continuous vacuuming.

Then, the bimetallic chitosan particles were synthesized by mixing a solution of secondary metal (copper) with Mg^0^-chitosan particles. The copper metal stock solution was prepared by dissolving CuCl_2_ in double distilled water. The bimetallic chitosan was prepared using copper bulk loadings of 1 wt.% by diluting the appropriate amount of the copper stock solution to 100 mL with double distilled water and then adding this solution to 10 g of fresh Mg^0^-chitosan particles according to the following redox reaction (1):

(1) $$\begin{array}{c} \text{Chitosan}-\text{M}{\text{g}}^{0}+\text{C}{\text{u}}^{2+}\to \text{M}{\text{g}}^{2+}+\text{Chitosan}-\text{C}{\text{u}}^{0}/\text{M}{\text{g}}^{0}. \end{array}$$

The samples were then shaken for 5 min, after which they were allowed to stand for 5 min at 25°C to enable the reduction of Cu^2+^ to Cu^0^. The resultant mixture was filtered by vacuum filtration through 0.2 *μ*m cellulose acetate filter paper. To get rid of the excess chemicals, the particles were washed with an excess amount of deoxygenated double distilled water and rinsed with ethanol and acetone before being dried at 50°C under vacuum overnight. Finally, the modified chitosan was stored under vacuum conditions for further use.

### 2.4. Experimental Design

Azo removal experiments with the prepared BCP were carried out as a batch test in a 100 mL flask while agitating on a shaker-incubator instrument (Pars Azma Co., Iran). The solution pH (3–10), BCP dosage (0.25–1.5 mg/L), pollutant concentration (50–200 mg/L), and contact time (1–60 min) were the selected variables in this step of the work. Each test consisted of preparing 50 mL of the azo solution with a desired initial concentration; the initial pH of the solution was adjusted by adding 0.1 N HCl and NaOH solutions. The shaking rate for all samples was 100 rpm. Aliquots were carefully withdrawn from the solution at various time intervals, and the solution absorbance was determined in the UV-visible range at the maximum absorption (*λ* = 483 nm) using a PuXi UV-vis spectrophotometer (TU-1900, China). Centrifugations performed on several samples showed that the aliquots were particle-free, and thus there was no need to carry out centrifugations for the taken aliquots. The percentage of dye removal and the adsorption capacity at equilibrium, *q*_*e*_ (mg/g), by BCP were calculated by using the following equations:

(2) $$\begin{array}{c} \text{OI}{\text{I}}_{\text{removal}}\left(\%\right)=\frac{C_{0}-C_{e}}{C_{0}}\times 100,\\ q_{e}=\frac{V}{M}\left(C_{0}-C_{e}\right), \end{array}$$

where *C*_0_ denotes the initial OII dye concentration (mg/L); *C*_*e*_ the OII dye concentration at equilibrium (mg/L); *V* the volume of the OII dye solution used (L); and *M* the mass of the adsorbent used (mg).

The kinetic and isotherm experiments of OII dye adsorption onto the BCP were carried out at designated concentrations (50–200 mg/L) and pH. After the adjustments, 1 mg/L of BCP was added, and the resultant suspension was stirred at 100 rpm for 1–60 min (except for isotherms experiment: 640 min) at 24°C in a temperature-controlled shaker incubator. Upon completion, the supernatant was pipetted out for a residual OII dye analysis. The results were kinetically and isothermally analyzed for fitness with models in order to determine the order and the rate constant of the OII dye adsorption onto BCP. All experiments were conducted in duplicate to ensure the reproducibility of the results, and the average values of the efficiency removal were reported. Control experiments containing no BCP were also prepared.

In order to examine the applicability of BCP for the treatment of wastewater containing azo dyes, an adsorption batch test was conducted using a river water sample spiked with the OII dye to 100 mg/L as wastewater. The amount of TOC was measured by using Shimadzu TOC-5000 Analyzer (Shimadzu Co., Japan).

### 2.5. Adsorption Nature

The morphology and the size of the BCP particles were determined using a transmission electron microscope (TEM) (FEI Tecnai G2 20S-TWIN, USA). The pH of the zero point charge (pH_zpc_) for BCP was also determined according to the method stated by Asgari et al. (2012) [17]. The probable degradation intermediates of OII were identified using a separate HPLC-MS (PerkinElmer Flexar SQ 300 MS, USA) analysis of the treated OII dye solution.

## 3. Results and Discussion

### 3.1. Characterization of BCP

**Figure 1:**
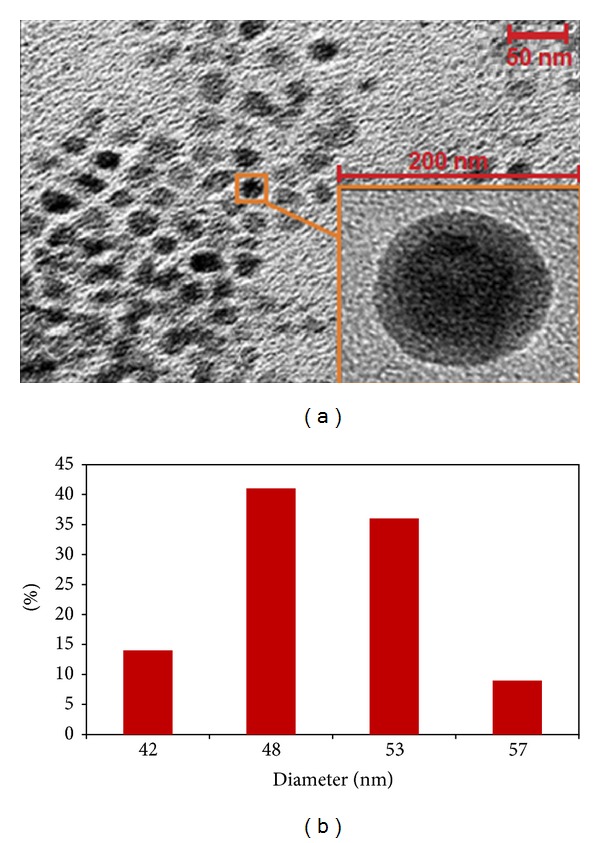
(a) TEM images of freshly prepared BCP; inset is TEM image of an individual Cu/Mg on BCP. (b) Size distribution of BCP.

The representative transmission electron microscope (TEM) images of the as-synthesized sample are shown in Figure 1. An overview image (Figure 1(a)) at low magnification illustrates that the sample entirely contains of individual Cu/Mg particles with a relatively same size distribution without the presence of larger particles or agglomerates. The Cu/Mg particles are considerably uniform in size and shape and mostly spherical. Typically, the BCP diameter ranges from 42 to 57 nm. The image of an isolated particle at higher magnification (inset in Figure 1(b)) shows the clear core-shell structure, giving additional evidence that the Cu/Mg particles are enwrapped by chitosan. Figure 1(b) shows the statistical information of the BCP size, which was obtained by measuring every particle on the same TEM photograph. It can be seen from Figure 1(b) that the column of 48 nm is the highest and that of 53 nm is the second highest. The percentages of the bimetal chitosan particles with diameters of 48 and 53 nm were 41% and 36%, respectively. This implies that there are approximately 77% particles having a diameter of 48–53 nm.

### 3.2. pH Effect and Adsorption Potential of BCP

**Figure 2:**
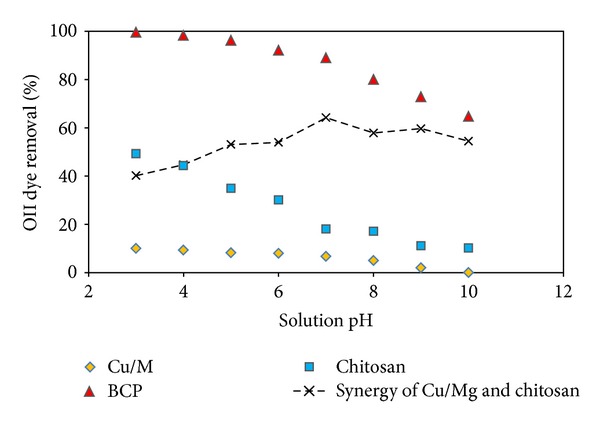
The synergy effect of Cu/Mg and chitosan during OII removal by BCP (OII concentration 200 mg/L, reaction time 10 min, and BCP and chitosan dose 1 mg/L).

**Figure 3:**
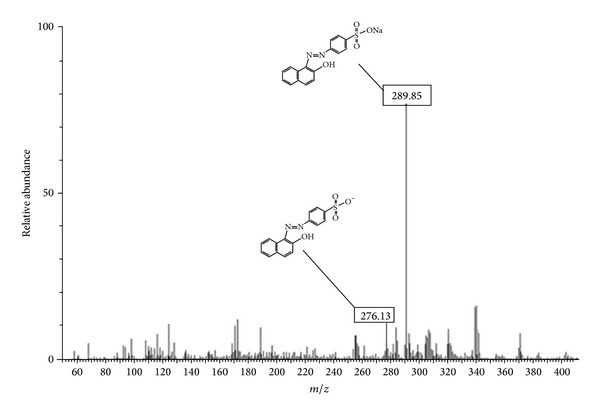
MS spectra of OII after treatment by BCP.

In order to provide supplementary evidence for the proposed nature of mechanisms involved in the OII removal by BCP, an experiment was conducted to remove OII by using Cu/Mg, chitosan, and BCP. As can be observed from Figure 2, the OII removal by Cu/Mg, chitosan, and BCP at pH 3 was attained at 10, 49.3, and 99.5%, respectively. The removal nature of dyes by using chitosan as previously reported by other researchers [18, 19] was adsorption. Only the bimetallic particles were applied for the dechlorination of contaminants [7, 20]; however, the azo dye removal by bimetallic particles has not been reported to date. Therefore, in order to identify the probable intermediate products of OII dye degradation by BCP, some supernatant of samples were analyzed by using the HPLC-MS. The HPLC-MS analysis of samples (Figure 3) showed the presence of two species: residual OII dye and protonated OII dye. Therefore, it can be concluded that the Cu/Mg could not degrade OII; however, it probably facilitated and synergized the adsorption of OII onto BCP. This also implies that the main approach in OII abatement by BCP can account for the adsorption process.

Here, the influence of pH on the removal of the OII dye using Cu/Mg, chitosan, and BCP is discussed in detail in order to obtain further insight into the adsorption process. The results illustrated in Figure 2 demonstrate that the OII dye removal by Cu/Mg, chitosan, and BCP is a strongly pH-dependent process. The average OII dye removal by BCP decreased from 99.5% to 79.9% when the solution pH increased from 3 to 8. The maximum adsorption of the OII dye was attained at pH 3 in accordance with previous reports [21–23]. The OII dye removal by BCP was significantly higher than the removal percentage achieved by the use of chitosan alone.

We offer some plausible reasons for the notable difference between the capabilities of BCP and chitosan to remove the OII dye.

(i) The Cu/Mg bimetal on the surface of chitosan plays an important role in the OII dye removal. For better understanding, we attempted to quantify the synergy of Cu/Mg and chitosan in the OII dye removal. The results are depicted in Figure 2. To accomplish this, the BCP potential toward the OII removal was calculated using the following equation:

(3) BCP  potential=[removal  in  BCP             –(adsorption  in  chitosan             +  removal  in  Cu/Mg)].

The rapid OII dye elimination by the Cu/Mg bimetal may be explained by mechanisms such as the formation of metal-hydride (M–H) complexes with copper and the dissociation of molecular hydrogen or other hydrogen sources on the second metal surface (Cu) [7–9], serving an important role in the OII dye removal. As previously reported, amine and hydroxyl groups are the main reactive groups of chitosan that contribute in the adsorption process [6] in the BCP-dye systems. The reduction of magnesium in the Cu/Mg bimetal induced H^+^ to an aqueous solution. In the presence of H^+^, the amino groups of chitosan became protonated (–NH_2_ → –NH_3_^+^); moreover, according to the MS spectra of OII (Figure 3), in the aqueous solution, the sulfonate groups in the OII dye (see Table 1) began to dissociate and were converted to anionic dye ions (Dye–SO_3_Na → Dye–SO_3_^−^ + Na^+^); the adsorption process then proceeded because of the electrostatic attraction between these two counter ions (–NH2 + Dye–SO_3_^−^ → –NH_3_^+^ + ^−^O_3_S–Dye). Therefore, OII removal by chitosan was promoted in the presence of Cu/Mg. In the other word, the chitosan and Cu/Mg bimetal had a synergistic effect on the OII abatement from a solution.

(ii) As observed from Table 3, the final pH of the solution after dye removal by BCP increased rapidly within 10 min, while, during the dye removal process by chitosan, the final pH of the solution (at the end of each experiment) remained similar to the initial value. This increase was attributed to the consumption of protons (H^+^) and the generation of hydroxide ions (OH^−^), as shown in the following equation:

(4) (Cu–Mg)+H2O+[OII] →(Cu–Mg2+)+OH−+[OII]–H.

The formation and precipitation of Mg(OH)_2_ during the treatment could have led to the coprecipitation of the OII dye; therefore, the BCP in the alkaline pH range was observed to be remarkably efficient for dye removal as compared to chitosan alone in this study and other previous studies [24, 25]. Thus, the modified chitosan, BCP, may be introduced as an efficient method for OII dye removal at all pH values, as the percentage of dye removal by the BCP at pH 3 and 10 was found to be 99.5 and 64.7, respectively.

(iii) As mentioned in the literature [26, 27], the Cu in the bimetal of Cu/Mg had an affinity to adsorbing some pollutants. Therefore, to some extent, the high efficiency of the BCP can account for dye adsorption by copper.

(iv) The relationship between the effects of the BCP in the OII dye adsorption and the solution pH can be explained by considering both the surface charge of the adsorbent and the dissociation constant (p*K*_*a*_) of the OII dye. Since the pH_zpc_ of BCP was 6.6, a positive charge developed on BCP surfaces at pH below pH_zpc_. The OII dye has p*K*_*a*_ of 1 (hydroxyl) and 10.6 (amino) [28]. Therefore, in an acidic solution, the hydroxyl group (AOH) present in the OII dye molecule dissociated into AO^−^. Accordingly, electrostatic attraction between the OII dye (anionic) molecules and the surface of the BCP (positively charged at pH below 6.6) was most likely the predominant adsorption mechanism. However, the reduced OII dye removal with an increased pH beyond 6 could be justified by an increase in the OH^−^ formation in the solution and the subsequent competition with the AO^−^ anions of the OII dye molecules for active adsorption sites on the surface of the BCP. The reduction of OII dye adsorption at alkaline solution pH (pH > pH_zpc_) could be related to a negative charge on the surface of the BCP, thereby activating the electrostatic repulsion of the anions for the adsorption on the adsorption sites onto the BCP. Abramian and El-Rassy [28] reported the maximum adsorption of the OII dye onto a porous titania aerogel, which occurred at an acidic solution pH.

### 3.3. Effect of BCP Dosage and Reaction Time

**Figure 4:**
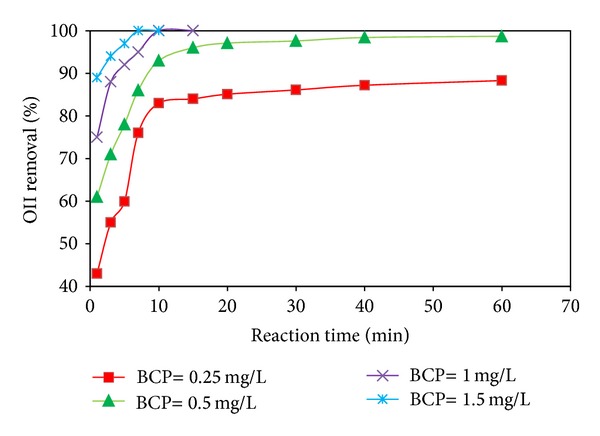
Effect of BCP dosage on OII removal as a function of reaction time (OII concentration 200 mg/L and pH 6).

The OII dye removal as a function of reaction time at various BCP doses (0.25–1.5 mg/L) was investigated and the results have been shown in Figure 4. Two important points arise from Figure 4. First, for all levels of BCP doses, the removal percentage improved with an increase in the reaction time, with the highest rate at the initial reaction times. This may be attributed to free adsorption sites available in the initial phases of the test [29], as well as to a higher mass transfer rate (due to a higher driving force) at initial reaction times where a higher concentration of OII dye is available for removal; thus, the rate of adsorption was observed to be greater. The second point is the increase in the OII dye removal process with an increase in the BCP dose, resulting in a shorter treatment time. According to Figure 4, around 55.4, 71.3, 88.6, and 94.2% of 200 mg/L OII dye were removed during the 3 min reaction time for BCP quantities of 0.25, 0.5, 1, and 1.5 mg/L, respectively. The degree of OII dye removal reached a maximum of 88.3 and 98.7% after 60 min when in contact with doses of 0.25 and 0.5 mg/L, respectively, whereas 100% removal efficiency was achieved for a 10 min reaction time in the presence of both BCP dosages of 1 and 1.5 mg/L. The enhancement of the OII dye removal as a function of the BCP dose was due to an increase in the available binding sites in the adsorbent [28] for OII dye molecules to be adsorbed, leading to the uptake of more molecules from the solution in high doses for a similar reaction time. This resulted in a greater efficiency for OII dye removal. Our findings are in agreement with those reported previously for the adsorption of the dye onto titania aerogel [28] and multiwalled carbon nanotubes [24], although we had an efficient dye removal process completed in a very short time, which was due to the different operational conditions and the adsorbent modifications made by the Cu/Mg bimetal. The high doses of BCP provided high amounts of Cu/Mg and more hydrogenation of azo dye consequently increasing the affinity of the dye to be adsorbed and the decolorization of the dye solution. Therefore, chitosan modified by Cu/Mg decreased the time required for a significant dye removal.

### 3.4. Isotherms Modeling

To obtain greater insight on the adsorption of OII onto BCP, the results of the equilibrium experiments were evaluated using the models of Langmuir, Freundlich, and Dubinin-Radushkevich (D-R).

The Freundlich isotherm assumes that the adsorption occurs on a heterogeneous surface, and the amount that is adsorbed increases infinitely with an increase in concentration. The Freundlich isotherm is given by [30]

(5) $$\begin{array}{c} \ln q_{e}=\ln K_{F}+\frac{1}{n}\ln C_{e}, \end{array}$$

where *K*_*F*_ denotes the Freundlich constant (mg/g) (mg/L)^−1/*n*^ and 1/*n* denotes the heterogeneity factor.

The Langmuir isotherm model assumes a monolayer adsorption onto a homogeneous surface where the binding sites have equal affinity and energy. The Langmuir isotherm is given by [31]

(6) $$\begin{array}{c} \frac{C_{e}}{q_{e}}=\;\frac{1}{K_{L}q_{\max}}+\frac{C_{e}}{q_{\max}}, \end{array}$$

where *q*_max⁡_ denotes the maximum adsorption capacity (mg/g) and *K*_*L*_ is the Langmuir constant (L/mg).

Another essential characteristic of the Langmuir isotherm can be expressed by the separation factor (*R*_*L*_), *R*_*L*_ = 1/1 + *K*_*L*_ *C*_*i*_ [31].

The D-R model (7) was also applied to express the adsorption isotherms [1]:

(7) $$\begin{array}{c} \ln q_{e}=\ln q_{m}-K_{\text{DR}}\varepsilon^{2}, \end{array}$$

where *q*_*m*_ denotes the maximum adsorption capacity (mg/g); *K*_DR_ a constant (mol^2^/kJ^2^); and *ε* the Polanyi potential (J/mol).

The information obtained from isotherm modeling is summarized in Table 4. The results revealed that the *R* ^2^ of the Langmuir isotherm was greater than that of the other models, indicating that the Langmuir isotherm better represented the adsorption of OII onto BCP. This result proposed that the adsorption of the OII occurred on a monolayer of the BCP surface. The conformity of the experimental data with the Langmuir model was in agreement with most of the previously published experiments [32, 33]. The maximum adsorption capacity of the OII onto BCP, obtained from the fitted Langmuir model, was 384.6 mg/g (see Table 4), which was greater than most of the adsorbents reported for adsorption of azo dyes [32, 33]. Moreover, the favorability of OII adsorption on BCP was further evaluated by using the dimensionless parameter, *R*_*L*_, which was derived from the Langmuir model. As shown in Table 4, the *R*_*L*_ values (0.11–0.33) for OII adsorption onto BCP are between 0 and 1, indicating that the adsorption process is favorable. Table 4 indicates that the value of the constant 1/*n* in the Freundlich model is greater than unity, which confirms the suitability [34] of BCP as an adsorbent for OII adsorption from wastewater. Further, results obtained from the evaluation of the D-R model (Table 4) suggest that the amount of free energy in the OII adsorption by BCP is 10.42 kJ/mol. The value of *E* lies in the range of 8–16 kJ/mol and indicates that chemisorption is the dominant process under the experimental conditions [35].

### 3.5. Kinetic Behavior

Information on adsorption kinetics is needed to select the optimum operating conditions for industrial applications [36] and is useful for determining the adsorption rate and thus the time needed to attain equilibrium. In order to analyze the adsorption kinetic behavior of the OII dye onto BCP, we used the adsorption reaction models (pseudo-first-order, pseudo-second-order, and Elovich model). The equations of the pseudo-first-order (8), pseudo-second-order (9), and Elovich model (10) can be written as follows:

(8) $$\begin{array}{c} q_{t}=q_{1}\left(1-\exp\left(k_{1}t\right)\right), \end{array}$$

(9) $$\begin{array}{c} q_{t}=\frac{t}{\left(1/k_{2}\;q_{2}^{2}\;\right)+\left(t/q_{2}\;\right)}, \end{array}$$

(10) $$\begin{array}{c} q_{t}=\frac{1}{\alpha }\ln\left(1+\alpha \beta t\right), \end{array}$$

where *q*_*t*_ denotes the adsorbate amount adsorbed at time *t* (mg/g), *q*_1_ and *q*_2_ indicate the theoretical values for the adsorption capacity (mg/g), *t* stands for the reaction time (min), and *k*_1_ and *k*_2_ denote the rate constants of the pseudo-first- and pseudo-second-order models, respectively, in (1/min) and (g/mg·min). In (10), *α* denotes the initial velocity due to d*q*/d*t* with *q*_*t*_ = 0 (mg/g·min) and *β* the desorption constant of the Elovich model (g/mg).

The coefficients of the kinetic equations were specified by nonlinear regression using the software Statistica 6.0 (Statsoft, USA), verifying its fit through the coefficient of determination (*R* ^2^) and the average relative error (ARE):

(11) $$\begin{array}{c} \text{ARE}\left(\%\right)=\frac{100}{n}\overset{n}{\underset{1}{\sum }}\frac{q_{e,\exp}-q_{e,\text{cal}}}{q_{e,\text{cal}}}, \end{array}$$

where *q*_*e*,exp⁡_ and *q*_*e*,cal_ denote the experimental values of adsorption capacity in time *t* and are obtained from kinetic models.

A summary of the information related to kinetic models is presented in Table 5. Based on Table 5, for three tested concentrations, the pseudo-first-order model did not show a good fit with the experimental data (*R* ^2^ < 0.95 and ARE > 5%). The pseudo-first-order model assumes that adsorption occurs because of a concentration difference between the dye surface and the solution. This occurs only during adsorption and is obtained when an external mass transfer coefficient controls the process [6]. This shows that the adsorption of the OII dye onto BCP was not controlled only by an external mass transfer coefficient. In other words, the pseudo-second-order and Elovich models showed a good fit with the experimental data (*R* ^2^ > 0.95 and ARE < 5%) (Table 5). The pseudo-second-order model had the same equation for internal and external mass transfer mechanisms [37] and suggested that adsorption under the studied conditions most likely depended on both the BCP and the OII dye and that chemisorption most likely controlled the overall adsorption rate [18, 34] of the OII dye onto BCP. The pseudo-second-order adsorption rate constants, *k*_2_, for three concentrations of 50, 100, and 200 mg/L of the OII dye are also shown in Table 5. The values of *k*_2_ decreased with an increase in the target pollutant concentration that indicated the enhanced mass transfer rate with an increased concentration gradient. The Elovich model is used when chemisorption occurs and the adsorption rate decreases with time because of the saturation of the adsorption sites on the surface [19]. In this case, a good fit with the pseudo-second-order and the Elovich models suggest that adsorption of the OII dye onto BCP occurs by internal and external mass transfer mechanisms and the adsorption process is of a chemical nature. Similar behavior was previously reported [19, 37].

### 3.6. Treatment of Simulated Wastewater

In order to assess the potential of BCP for the treatment of wastewater containing azo dyes, we used a river water sample spiked with the OII dye to 100 mg/L as wastewater. The experiment was conducted at pH level as per natural water (ca. 6.82) with BCP concentrations of 1 mg/L and contact time of 20 min. The main parameters of the wastewater sample before and after treatment with BCP are presented in Table 6. As seen in Table 6, a very low amount of BCP (1.5 mg/L) at a relatively short reaction time (20 min) could completely remove the OII dye and improve some of the other characteristics of the treated wastewater. An important point found in Table 6 implied a reduction in some parameters during the treatment. These findings confirm the capability of BCP in treating azo wastewater. Considering that BCP can be simply prepared and applied at currently active wastewater treatment facilities, it implies that the BCP treatment process presents an efficient, low-cost, and viable technology for the treatment of dye wastewater.

## 4. Conclusions

The adsorption of the OII dye by the developed adsorbent, BCP, was tested under various operational variables. It was found that in a wide range of pH levels, the removal efficiency by BCP was remarkable; this presented a significant advantage for the practical application of the BCP. The results showed that the coating of a small amount of Cu/Mg bimetallic particles on the surface of chitosan could promote the dye removal, where the OII dye removal by chitosan alone and bimetal-chitosan was attained to be 49 and 99.5%, respectively. The experimental data could be better interpreted by the Langmuir model, and the maximum adsorption capacity of BCP for OII was demonstrated to be 384.6 mg/g. The results revealed that the pseudo-second-order and Elovich models fit the kinetic experimental data and that chemisorption was the dominant process for dye removal by BCP under the experimental conditions. Moreover, a significant degree of treatment was achieved for OII during the treatment of the simulated wastewater. Accordingly, it may be concluded that the developed BCP is an efficient method for the decolorization of azo dyes.

## Figures and Tables

**Table 1:** Main properties of OII used in this study.

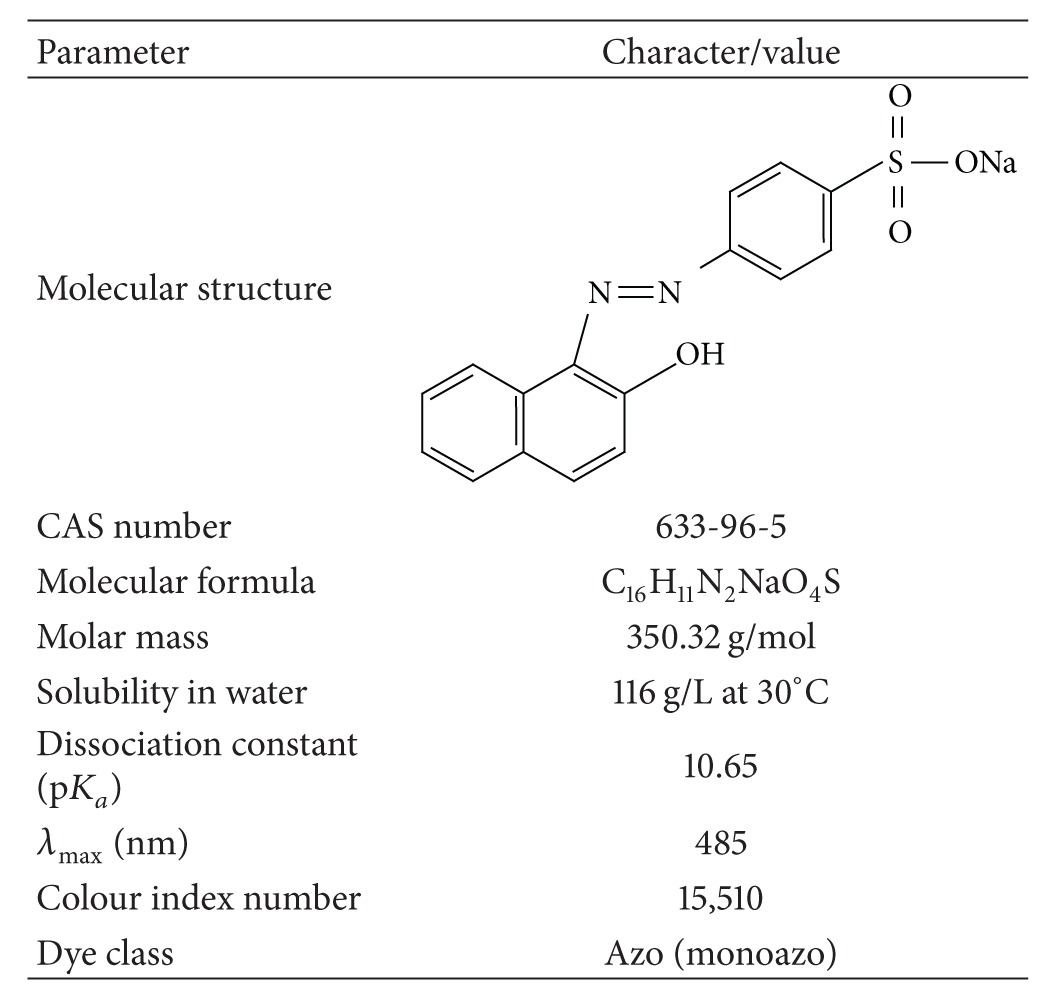

**Table 2:** Main characteristics of BCP adsorbent used in this study.

Parameter	Unit	Value
BET	m^2^/g	12.69
Total pore volume (*P*/*P*_0_ = 0.990)	cm^3^/g	0.198
Mean pore diameter	nm	49
Pores structure	—	Meso- and macroporous
pH_zpc_	—	6.6
Particle size	nm	42–57

**Table 3:** Variation of solution pH during OII removal by chitosan and BCP (OII concentration 200 mg/L, reaction time 10 min, and BCP and chitosan dose 1 mg/L).

Initial solution pH	Final pH	
	Chitosan	BCP
3	3.03	5.26
4	4.09	6.61
5	5.08	7.42
6	6.03	8.42
7	7.08	9.53
8	8.08	9.85
9	9.1	10.8
10	10.02	11

**Table 4:** Results of OII dye adsorption isotherm modeling.

Isotherm	Unit	Information
Langmuir model		*C*_*e*_/*q*_*e*_ = 1/*K*_*L*_ *q*_max⁡_ + *C*_*e*_/*q*_max⁡_
Plot	—	(*C*_*e*_/*q*_*e*_) versus C_e_
Fitted model	—	*C*_*e*_/*q*_*e*_ = 0.0026 + 0.039*C*_*e*_
*q*_max⁡_	mg/g	384.6
*K*_*L*_	L/mg	0.06
*R* ^2^	—	0.998
*R*_*L*_ = 1/1 + *K*_*L*_ *C*_*i*_	—	0.11–0.33
Freundlich model		ln *q*_*e*_ = ln *K*_*F*_ + 1/*n* ln C_e_
Plot	—	ln *q*_*e*_ versus ln C_e_
Fitted model	—	ln *q*_*e*_ = 3.85 + 0.4 ln C_e_
*K*_*f*_	—	46.9
1/*n*	mg/g (L/mg)^1/*n*^	2.48
*R* ^2^	—	0.984
D-R model		ln⁡⁡*q*_*e*_ = ln⁡⁡*q*_*m*_ − *K*_DR_ *ε* ^2^
Plot	—	ln *q*_*e*_ versus *ε* ^2^
Fitted model	—	ln⁡⁡*q*_*e*_ = 3.53 − 0.0046*ε* ^2^
*K*_DR_	mol^2^/kJ^2^	0.0046
*E* = 1/(2*K*_DR_)^0.5^	kJ/mol	10.42
*R* ^2^	—	0.979

**Table 5:** Kinetic details of OII dye adsorption onto BCP.

Model	Pseudo-first order			Pseudo-second order			Elovich		
Plot	(*t*/*q*_*t*_) versus *t*			(*t*/*q*_*t*_) versus *t*			*q*_*t*_ versus ln *t*		
Conc.	50	100	200	50	100	200	50	100	200
Fitted model	ln⁡(*q*_*e*_ − *q*_*t*_) = 1.079 − 0.075*t*	ln⁡(*q*_*e*_ − *q*_*t*_) = 1.09 − 0.045*t*	ln⁡(*q*_*e*_ − *q*_*t*_) = 2.309 − 0.025*t*	*t*/*q*_*t*_ = 0.078 + 0.066*t*	*t*/*q*_*t*_ = 0.053 + 0.051*t*	*t*/*q*_*t*_ = 0.037 + 0.028*t*	*q*_*t*_ = 0.078 + 51.26ln⁡*t*	*q*_*t*_ = 0.063 + 103.72ln⁡*t*	*q*_*t*_ = 0.981 + 146.7ln⁡*t*
*R* ^2^	0.885	0.882	0.875	0.995	0.991	0.988	0.993	0.985	0.980
Constant	*k*_1_ = −0.075	*k*_1_ = −0.045	*k*_1_ = −0.025	*k*_2_ = 0.056	*k*_2_ = 0.049	*k*_2_ = 0.021	*β* = 51.26	*β* = 103.72	*β* = 146.7
*q*_*e*_ (*q*_*e*,cal_)	2.94	2.97	10.06	15.15	19.6	35.7	*α* = 0.0195	*α* = 0.0097	*α* = 0.0068
*q*_*e*_ (*q*_*e*,exp⁡_)	34.1	42.1	57.1	16.4	22.1	36.1	34.1	34.1	34.1
ARE (%)	46.70	49.34	55.20	3.11	4.08	4.88	4.15	4.46	4.90

All units as described in Section 3.5.

**Table 6:** The quality of simulated wastewater before and after treatment with BCP (OII concentration 100 mg/L, pH 6.8, contact time 20 min, and BCP dosage 1 mg/L).

Wastewater parameter	Unit	Value	
		Raw wastewater	BCP-treated wastewater
OII dye	mg/L	100	<3
TOC	mg/L	54.9	52.1
pH	—	6.82	7.97
Temperature	°C	22	23
Turbidity	NTU	5	3
Nitrate	mg/L	12	4
UV_254_ absorption	Absorbance (1/cm)	0.15	0.005
EC	*μ*S/cm	432	428
